# Inhibins regulate peripheral regulatory T cell induction through modulation of dendritic cell function

**DOI:** 10.1002/2211-5463.12555

**Published:** 2018-12-11

**Authors:** Marisol de la Fuente‐Granada, Roxana Olguín‐Alor, Sandra Ortega‐Francisco, Laura C. Bonifaz, Gloria Soldevila

**Affiliations:** ^1^ Departamento de Inmunología Instituto de Investigaciones Biomédicas UNAM Mexico City Mexico; ^2^ Laboratorio Nacional de Citometría de Flujo Instituto de Investigaciones Biomédicas UNAM Mexico City Mexico; ^3^ Unidad de Investigación Médica en Inmunoquímica Instituto Mexicano del Seguro Social Centro Médico Nacional Siglo XXI Mexico City Mexico

**Keywords:** dendritic cells, inhibins, peripheral tolerance, Tregs

## Abstract

We have previously reported that the absence of inhibins results in impaired dendritic cell (DC) maturation and function, leading to decreased T cell activation and diminished delayed‐type hypersensitivity responses. Here, we investigated the role of inhibins in peripheral regulatory T cell (Treg) induction *in vitro* and *in vivo*. Inhibin deficient (Inhα^−/−^) mice showed an increased percentage of peripherally induced Tregs in colonic lamina propria and mesenteric lymph nodes, compared to Inhα^+/+^ mice, which correlated with increased expression of PD‐L1 in CD103^+^ and CD8α^+^ DCs. Lipopolysaccharide‐stimulated bone marrow‐derived and *ex vivo* spleen‐ and lymph node‐purified CD11c^+^ Inhα^−/−^ DCs induced higher Tregs *in vitro*. Moreover, *in vivo* anti‐DEC205‐ovalbumin (OVA) DC targeting of mice with adoptively transferred OVA‐specific T cells showed enhanced induced peripheral Treg conversion in Inhα^−/−^ mice. These data identify inhibins as key regulators of peripheral T cell tolerance.

**Abbreviations**
BMDC: bone marrow‐derived dendritic cell
BMP: bone morphogenetic protein
cDC: conventional dendritic cell
CT: cholera toxin
DC: dendritic cell
dLN: draining lymph node
FoxP3: forkhead box P3
FACS: fluorescence‐activated cell sorting
IL: interleukin
LP: lamina propria
LPS: lipopolysaccharide
mDC: migratory dendritic cell
MFI: mean fluorescence intensity
MHC‐II: major histocompatibility complex class II
MLN: mesenteric lymph node
OVA: ovalbumin
PLN: peripheral lymph node
pTreg: peripherally induced Treg
RA: retinoic acid
rDC: resident dendritic cell
TCR: T cell receptor
TGFβ: transforming growth factor β
Th3: T helper 3
Tr1: type 1 regulatory T
Treg: regulatory T cell
tTreg: thymic regulatory T cell
WT: wild‐type

Regulatory T cells (Tregs) play a key role in central and peripheral T cell tolerance by preventing the development of autoimmunity and restraining inflammatory immune responses to pathogens that may result in immunopathology. The balance between effector and regulatory T cells is critical for the maintenance of homeostasis (reviewed in 1).

Tregs are a subset of CD4^+^ T cells characterized by a high expression level of CD25 (interleukin (IL)‐2α chain receptor) and forkhead box P3 (FoxP3), a transcription factor considered the master regulator of Treg development and function 2. Their ability to suppress several immune cell responses has become increasingly relevant to understanding and treating several diseases and inflammatory responses 3. Two major Treg subsets have been identified, those originating in the thymus, referred to as thymic Tregs (tTregs), and those induced in peripheral tissues from naïve T cells, referred to as peripheral Tregs (pTregs) (reviewed in 4). Both populations share some phenotypic markers including FoxP3, CD25, GITR and CTLA‐4, although other markers, such as neuropilin 1, CD73 and Helios, have been proposed as specific for tTregs 5, 6. In addition, the signaling events needed to induce pTregs are different from those required for tTreg differentiation; transforming growth factor β (TGFβ) is a required cytokine for FoxP3 induction in pTregs, as well as low levels of T cell receptor (TCR) activation and low costimulatory signals. In contrast, tTregs require strong TCR and costimulatory signals and the presence of γ chain cytokines, such as IL‐2 and/or IL‐7. These different requirements are associated with the transcriptional regulation of the FoxP3 gene in tTregs *versus* pTregs 7. Concerning the functional relevance of Treg subpopulations, tTregs have been shown to play a crucial role in the control of autoimmune diseases 8, while pTregs appear to be more relevant in restraining immunopathology after an immune response and in the context of intestinal homeostasis 9 (reviewed in 10). However, both tTregs and pTregs have been shown to be necessary to prevent colitis, showing a non‐redundant role in the maintenance of peripheral tolerance 11.

In addition to FoxP3^+^ Tregs, other regulatory T cell subsets can be induced from naïve T cells, such as type 1 regulatory T (Tr1) cells and T helper 3 (Th3) cells (reviewed in 12). Compared with Tregs, Tr1 and Th3 cells normally do not express CD25 or FoxP3 13, 14. Tr1 cells are characterized by the expression of CD49b, LAG3 and the production of IL‐10; their differentiation is favored under suboptimal antigen stimulation in the presence of IL‐10 15, 16. On the other hand, Th3 cells are characterized by the production of TGFβ1 and the expression of CD69^+^ and LAP^+^ 14, 17.

Dendritic cells (DCs) are a heterogeneous group of professional antigen presenting cells that originate in the bone marrow, principally from myeloid progenitors that differentiate into Pre‐DCs. Pre‐DCs seed peripheral tissues, where they complete their differentiation to DCs, in the lymph node, where they are known as resident DCs (rDCs), or in non‐lymphoid tissues, where they are known as migratory DCs (mDCs) 18, 19. Both conventional DC (cDC) subsets can be identified in lymph nodes as CD11c^hi^MHC‐II^med^ and CD11c^med^MHC‐II^hi^ for rDCs or mDCs, respectively 19. DCs play an important role in peripheral tolerance through several mechanisms including clonal deletion, anergy and regulation. In homeostasis, DCs capture self‐antigens and present them to naïve T cells, preventing the activation of self‐reactive clones and favoring the induction of Tregs and T cell anergy. In this context, murine cDCs can be subdivided into two main subtypes that are considered independent cDC lineages: type 1 DCs (cDC1) for CD8α^+^ rDCs and CD103^+^ mDCs, and type 2 DCs (cDC2) for CD4^+^/CD11b^+^ rDCs and CD11b^+^ mDCs (reviewed in 20). CD103^+^ mDCs in mesenteric lymph node (MLN) are considered as tolerogenic DCs due to their low levels of costimulatory molecules (CD40, CD80 and CD86), high levels of coinhibitory molecules (PD‐L1 and PD‐L2) and the expression of IL‐10, retinoic acid (RA) and TGFβ, which can lead to Tr1 and FoxP3^+^ pTreg induction 21, 22. In addition, CD8α^+^ rDCs have also shown tolerogenic potential through TGFβ production, and targeting antigen to CD205 (DEC205), leading to clonal deletion 23 and Treg differentiation 24.

The TGFβ family comprises several structurally related proteins, including TGFβ, bone morphogenetic proteins (BMPs), activins and inhibins 25. Inhibins and activins were first characterized as hormones 26 and are currently known to be involved in several immunological processes 27. The canonical signaling pathway of this family is highly conserved and is shared among TGFβ, BMPs and activins. Briefly, dimeric ligands bind their serine/threonine kinase receptors (type I and II) and lead to phosphorylation of receptor SMADs, which heterodimerize with the common SMAD and translocate to the nucleus thereby regulating gene expression 28. Several mechanisms have been proposed to explain the antagonistic effect of inhibins on activin‐mediated functions (reviewed in 29); inhibins are known to bind type II receptors through their β subunit and TGFβ type III coreceptor (TβRIII) through their α subunit, thus inhibiting the recruitment of type I receptor to the tertiary complex, interfering with SMAD‐dependent signaling. Consequently, inhibins were considered non‐signaling molecules; however, several reports support the possibility that inhibins may signal through a different receptor, which has not been identified to date (reviewed in 30). This is supported by evidence showing that inhibins do not always antagonize activin functions. Specifically, inhibins and activins were shown to similarly control specific checkpoints during T cell development 31; in addition, our group has shown that inhibins can regulate tTreg cell differentiation by controlling medullary/cortical thymic epithelial cell differentiation and DC maturation within the thymus 32. Moreover, in recent work, we have demonstrated that the absence of inhibins in DCs results in an impaired maturation, characterized by low expression of major histocompatibility complex class II (MHC‐II) and costimulatory molecules, as well as alterations in migration and, more importantly, diminished ability to initiate T cell responses, such as *in vitro* proliferation of allogeneic CD4^+^ T cells and delayed‐type hypersensitivity responses 33.

## Materials and methods

### Mice

Inhibin α heterozygous mice (Inhα^+/−^) in C57BL/6 background were donated by M. Matzuk (Baylor College of Medicine, Houston, TX, USA) and have been previously described 34. FoxP3^EGFP^ knock‐in mice (B6.Cg‐Foxp3tm2Tch/J), CD45.1 and OT‐II transgenic mice in C57BL/6 background were purchased from The Jackson Laboratory (Bar Harbor, ME, USA). Mice were intercrossed to generate Inhα^+/+^FoxP3^EGFP^, Inhα^−/−^ FoxP3^EGFP^ and CD45.1^+^OT‐II^+^ mice. Mice were bred and maintained in the animal facility of the Instituto de Investigaciones Biomédicas (IIB, UNAM, México), in specific pathogen free conditions, according to ethics guidelines. The study was approved by the Comité para el Cuidado y Uso de Animales de Laboratorio (CICUAL) of the IIB. For all experiments, 4‐week‐old female mice were used.

### Preparation of lymphocyte suspensions from colonic lamina propria, mesenteric lymph node, peripheral lymph nodes or spleen

Lymphocytes from colonic lamina propria (LP) were isolated using modified methods previously described 35. Briefly, the gut was flushed with PBS, opened longitudinally and colon was cut into 5 mm pieces. The tissue was incubated in calcium‐ and magnesium‐free HBSS containing 2 mm EDTA and 1 mm dithiothreitol (Sigma‐Aldrich, St. Louis, MO, USA) for 30 min at 37 °C in a shaking incubator. The remaining tissue was washed with PBS, and incubated for 30 min more at 37 °C in RPMI supplemented with 100 U·mL^−1^ collagenase IV (Thermo Fisher Scientific, Waltham, MA, USA). Cell suspensions were filtered with 150 μm nylon mesh. MLNs, peripheral lymph node (PLN) and spleen were harvested, mechanically disaggregated, and filtered to obtain a cell suspension. In the case of spleen, erythrocytes were lysed with Ammonium‐Chloride‐Potassium lysing buffer. Cells were resuspended in fluorescence‐activated cell sorting (FACS) buffer for phenotype analysis or PBS for FACS of naïve T cells.

### Preparation of DCs from MLN, PLN or spleen

DCs were obtained after collagenase digestion from MLN, PLN and spleen, as previously described 33. Cells were resuspended in FACS buffer for phenotype analysis. CD11c^+^ magnetic‐activated cell sorting‐enriched DCs, lipopolysaccharide (LPS)‐stimulated (mCD11c^+^) or not (iCD11c^+^), were used in the functional assays.

### Flow cytometry

For phenotypic analysis, single cell suspensions were stained as previously described 36. For *ex vivo* Treg cell analysis, anti‐CD25‐PECy5, anti‐Helios‐FITC, anti‐CD8‐PE (from Biolegend, San Diego, CA, USA), anti‐CD4‐APC‐AF750 (from Thermo Fisher Scientific), and anti‐FoxP3‐APC (from eBiosciences, San Diego, CA, USA) were used. For *in vitro* induced Treg analysis, Zombie Aqua fixable dye, anti‐CD4‐APC and anti‐CD25‐PECy5 from Biolegend were used. For *ex vivo* DC analysis, cells were blocked with purified anti‐CD16/32, followed by staining with Zombie Aqua, anti‐I‐A/I‐E‐AF488, anti‐CD11c‐AF700, anti‐CD80‐PECy5 (from Biolegend), anti‐CD3‐PE, anti‐TER119‐PE, anti‐CD11b‐VF450, anti‐CD86‐APC, anti‐CD8‐PECy7 (from Tonbo Biosciences, San Diego, CA, USA), anti‐CD19‐PE, anti‐CD49b‐PE, streptavidin‐APCCy7 (from BD Biosciences, San Jose, CA, USA), anti‐CD103‐biotin and anti‐PD‐L1‐PerCP‐eFluor710 (from eBiosciences) were used.

For *in vivo* transfer experiments, anti‐CD45.1‐AF700, anti‐CD4‐FITC, anti‐CD25‐PECy5, streptavidin‐BV605 (from Biolegend), anti‐Vβ5‐biotin (from BD Biosciences), and anti‐FoxP3‐APC (from eBiosciences) were used for staining ovalbumin (OVA)‐specific T cells.

Samples were acquired in an Attune Acoustic Focusing Flow Cytometer (Thermo Fisher Scientific) and analyzed using flowjo 10.0 software (Tree Star Inc., Ashland, OR, USA).

### Generation of bone marrow‐derived DCs

Bone marrow derived DCs (BMDCs) were obtained from femurs and tibias of mice, as previously described 33. Cells were resuspended in RPMI supplemented with 10% FBS, 100 U·mL^−1^ penicillin and 100 μg·mL^−1^ streptomycin, and differentiated with granulocyte–monocyte colony‐stimulating factor. After 5 days of culture, mature BMDCs (mBMDCs) were obtained after stimulation with 1 μg·mL^−1^ *Escherichia coli* 0111:B4 LPS for 24 h. At day 6, non‐adherent cells were harvested, and CD11c^+^ cells were purified by magnetic‐activated cell sorting and used for further experiments.

### Treg cell induction

For *in vitro* cultures, naïve CD4^+^CD25^−^CD44^low^CD62L^hi^FoxP3‐GFP^−^ T cells were sorted from spleen and PLN from FoxP3^EGFP^ mice and cocultured with either CD11c^+^ BMDCs or spleen and PLN CD11c^+^ DCs, at different DC : Tnaïve ratios (1 : 1, 1 : 2, 1 : 4, 1 : 10, 1 : 20). Cultures were stimulated with 0.1 μg·mL^−1^ anti‐CD3 (Tonbo) and 0.25 ng·mL^−1^ TGFβ (R&D systems, Minneapolis, MN, USA). Expression of FoxP3 and CD25 was evaluated after 5 days by flow cytometry.

For *in vivo* peripheral Treg induction, CD4^+^CD25^−^ T cells were sorted from PLN and spleen of OT‐II × CD45.1 mice; 4 × 10^6^ cells were transferred intravenously to CD45.2 Inhα^+/+^ or Inhα^−/−^ mice. After 24 h, intradermal immunization with anti‐DEC205‐OVA, anti‐DEC205‐OVA+cholera toxin (CT), OVA or OVA+CT was performed in the mouse ears. Seven days after immunization, pTregs were analyzed as CD25^+^FoxP3^+^ within the population of transferred OT‐II cells (CD4^+^CD45.1^+^Vβ5^+^) in single cell suspensions obtained from draining lymph nodes (dLN).

### Statistical analysis

Data are presented as means ± SEM. The significance of results was calculated by paired or unpaired, one or two‐tailed Student's *t* test, utilizing prism 6 statistical software (GraphPad Software, La Jolla, CA, USA) *P* values < 0.05 were considered as statistically significant. *P* values > 0.05 and < 0.1 were considered as trends.

## Results and discussion

### Peripheral Tregs are increased in the absence of inhibins

**Figure 1:**
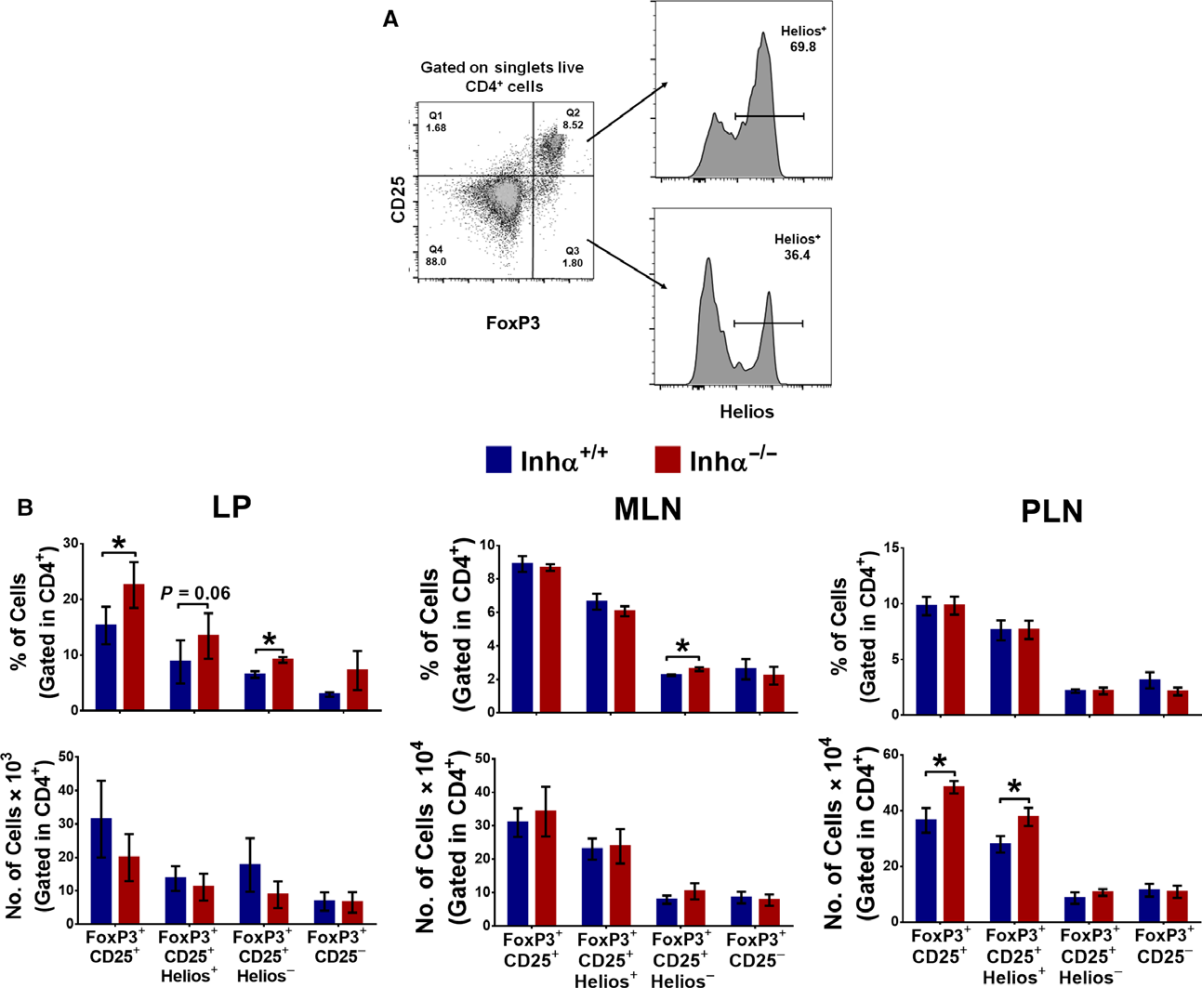
Tregs are incremented in the periphery in the absence of inhibin. Inhα^+/+^ and Inhα^−/−^ mice were analyzed for Tregs (CD4^+^CD25^+^FoxP3^+^), thymic (Helios^+^) or peripheral (Helios^−^). (A) Gate strategy for Treg analysis. (B) Frequency (top) and number (bottom) in colonic lamina propria (LP) (left), mesenteric lymph node (MLN) (center), and peripheral lymph node (PLN) (right). Mean ± SEM, *n* = 3–5 mice. Statistical significance was determined by two‐tailed unpaired Student's *t* test. *P ≤ 0.05.

To investigate whether inhibins play a role in the induction of Tregs in the periphery, we first evaluated Treg cell subpopulations from the Inhα^−/−^ or Inhα^+/+^ mice. Inhα^−/−^ is an α subunit null mouse where neither inhibin A nor inhibin B can be synthesized 34. As shown in Fig. 1, in the absence of inhibins, the numbers of CD25^+^FoxP3^+^ Tregs were significantly increased in PLN, specifically those Tregs expressing Helios, which correlates with our previous report showing enhanced tTreg development in Inhα^−/−^ mice 32. However, when we evaluated Treg subpopulations in MLN and colonic LP, we found an increased frequency of CD25^+^FoxP3^+^Helios^−^ Tregs which, under homeostatic conditions, are considered pTregs 37. These data suggest that inhibins regulate *de novo* generation, maintenance or recruitment of pTregs in the gut mucosa under homeostatic conditions. As the gut microenvironment provides a continuous stimulation from commensal bacteria and dietary antigens, this mucosa is particularly prone to tolerance induction by means of production of anti‐inflammatory cytokines (IL‐10, TGFβ), which promote Tr1 and pTreg conversion, while the production of RA by CD103^+^ DCs induced FoxP3 expression and gut homing molecules CCR9 and α4β7 integrins, which retain Tregs in the intestinal mucosa 10. Indeed, experiments using ‘depletion of regulatory T cell’ (DEREG) mice revealed that the constitutive presence of Tregs is required for the prevention of autoimmune inflammation and colitis 38.

### Mesenteric Inhα^−/−^ CD103^+^ DC display increased levels of PD‐L1

**Figure 2:**
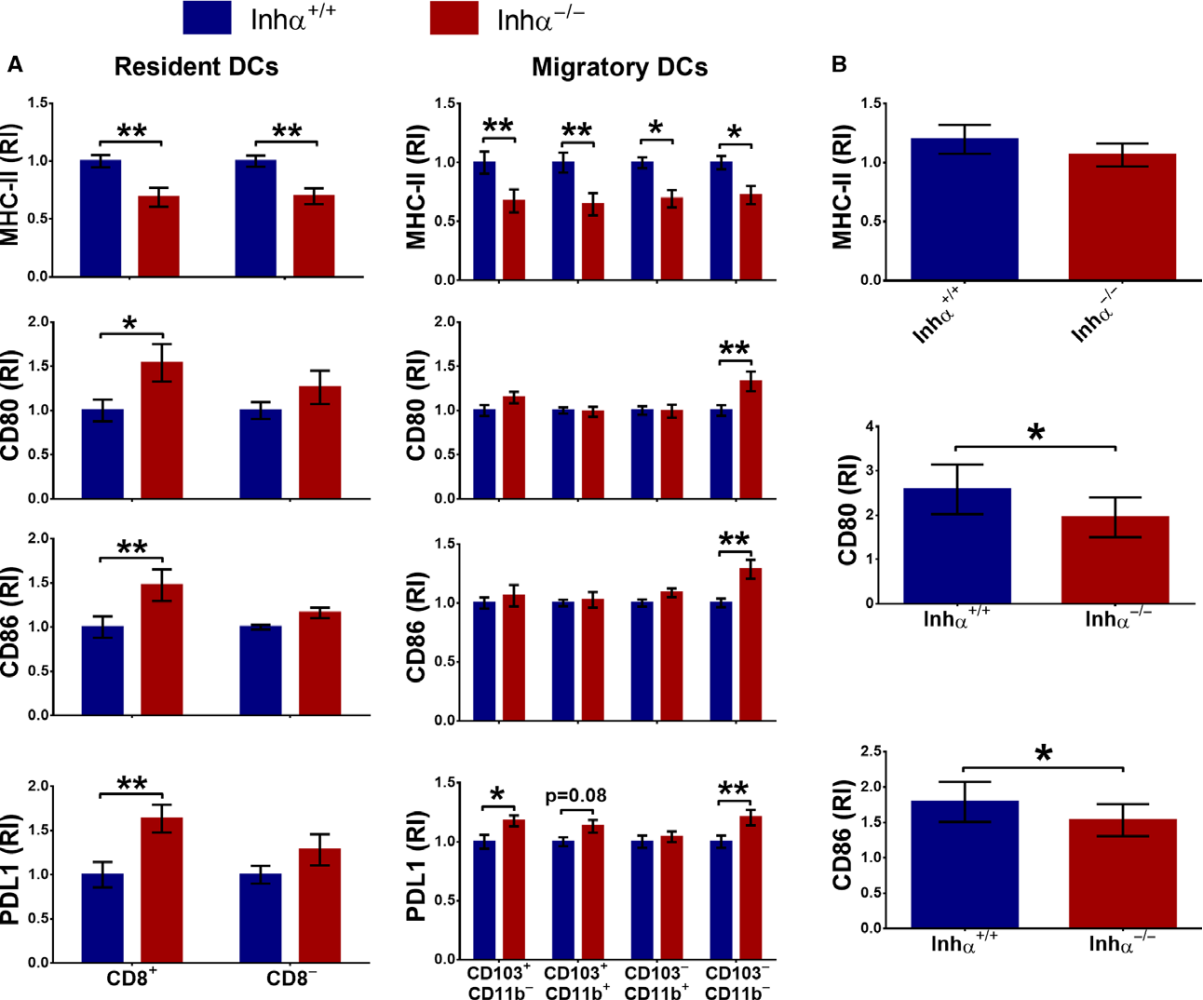
Inhα^−/−^ DC subsets have differential expression of MHC‐II, CD80 and PD‐L1 in MLN compared to Inhα^−/−^. Inhα^+/+^ and Inhα^−/−^ mice were analyzed for cDC subpopulations in MLN. (A) MHC‐II, CD80, CD86 and PD‐L1 expression within the resident DC (Lin^−^CD11c^hi^MHC‐II^+^CD8^+^, Lin^−^CD11c^hi^MHC‐II^+^CD8^−^) (left) and migratory DC (Lin^−^CD11c^+^MHC‐II^hi^CD103^+^CD11b^−^, Lin^−^CD11c^+^MHC‐II^hi^CD103^+^CD11b^+^, Lin^−^CD11c^+^MHC‐II^hi^CD103^−^CD11b^+^, Lin^−^CD11c^+^MHC‐II^hi^CD103^+^CD11b^−^) (right) subpopulations. (B) Analysis of MHC‐II, CD80 and CD86 in LPS‐stimulated splenic CD11c^+^ DC. Bar graphs represent relative expression of mean fluorescence intensity (MFI) compared to unstimulated splenic CD11c^+^ Inhα^+/+^ DCs. Relative expression was calculated as the ratio: MFI of LPS‐stimulated DCs/MFI of unstimulated DCs, for both Inhα^+/+^ and Inhα^−/−^ DCs. Mean ± SEM, *n* = 5 mice. Statistical significance was determined by the two‐tailed unpaired Student's *t* test. *P ≤ 0.05, **P ≤ 0.01.

We have recently reported that, in the absence of inhibins, DCs showed impaired maturation after *in vitro* LPS stimulation, which correlated with reduced capacity to induce CD4^+^ T cell proliferation *in vitro* and lower delayed‐type hypersensitivity responses *in vivo* 33. This ‘semi‐mature’ phenotype has been associated with the ability of DCs to promote tolerogenic responses including FoxP3^+^ Treg generation 39. To understand whether the increased pTregs observed in MLN and LP of Inhα^−/−^ mice were related to differences in MLN DC subpopulations, we analyzed the frequency and phenotype of DC subpopulations as shown in Fig. S1. We analyzed resident and mDCs, based on their expression of MHC‐II and CD11c, as CD11c^hi^MHC‐II^lo^ and CD11c^lo^MHC‐II^hi^, respectively. To further analyze DC subsets, we used CD8α to discriminate CD8α^+^ and CD8α^−^ rDCs, and for mDCs we used CD11b and CD103 to discriminate the following subpopulations: CD103^+^CD11b^−^, CD103^+^CD11b^+^ and CD11b^+^CD103^−^. A minor subpopulation, CD11b^−^CD103^−^, can also be observed; however, this subset has not been further characterized 40. Frequency and numbers of DC subsets analyzed were not altered in the absence of inhibins (not shown); however, Inhα^−/−^ DCs in MLN showed a diminished expression of MHC‐II in all DC subsets (Fig. 2A, upper graphs), similarly to our previous report showing lower MHC‐II expression on Inhα^−/−^ epidermal Langerhans cells 33. Interestingly, when we evaluated the expression of costimulatory/inhibitory molecules in MLN DC subsets we found a significantly increased expression of the coinhibitory molecule PD‐L1 in CD8α^+^ rDCs and in CD103^+^CD11b^−^ mDCs and a trend towards an increase of PD‐L1 in CD103^+^CD11b^+^ mDCs from Inhα^−/−^ mice. These CD103^+^ DC subpopulations have been reported to play a key role in tolerance induction in the gut, as they produce high levels of RA and TGFβ, which are key mediators of FoxP3 induction in the intestinal microenvironment 41, 42. In fact, it has been previously shown that CD103^+^CD11b^−^PD‐L1^hi^ DC are high inducers of pTregs 43, in agreement with the reported effect of PD‐L1 during Treg conversion from naïve T cells by immature DCs *in vitro* 44.

Despite the lower expression of MHC‐II, we observed an increase in CD80 and CD86 in CD8α^+^ rDCs and CD103^−^CD11b^−^ mDCs. In this context, CD80 and CD86 do not exclusively act as costimulatory molecules, as they can bind coinhibitory receptors such as CTLA‐4 and PD‐L1 with high affinity, favoring tolerance induction, by competing with costimulatory receptors (CD28) for T cell activation and inhibiting T cell proliferation 45, 46. Interestingly, a recent report has shown that expression of PD‐L1 can bind CD80 *in cis* on the same cell, blocking the binding of CD80 to its ligand 47. Therefore, coexpression of these molecules *in vivo* could promote a tolerogenic response.

As we observed an increase in ‘tolerogenic’ DCs in MLN of Inhα^−/−^ mice, we next evaluated whether spleen DCs were prone to differentiate into tolerogenic DCs in the absence of inhibins. As shown in Fig. 2B, LPS‐stimulated *ex vivo* Inhα^−/−^ CD11c^+^ splenic DCs showed decreased upregulation of MHC‐II and CD80 in comparison with Inhα^−/−^ counterparts (Fig. 2B). In summary, the tolerogenic phenotype of Inhα^−/−^ DCs may explain the enhanced pTreg generation in MLN. Alternatively, we cannot exclude an intrinsic effect of inhibins on T cells, since Inhα^−/−^ T cells appear to express different levels of TβRIII compared to Inhα^+/+^ T cells in response to TCR stimulation (S. Ortega‐Francisco, M. de la Fuente‐Granada, R. Olguín‐Alor, L. C. Bonifaz & G. Soldevila, manuscript in preparation). In this context, TβRIII acts as a coreceptor that potentiates TGFβ‐mediated signals 48 and most recently, our group has shown that it promotes Treg induction *in vitro* 36.

### Inhibins regulate DC‐mediated induction of Tregs *in vitro*

**Figure 3:**
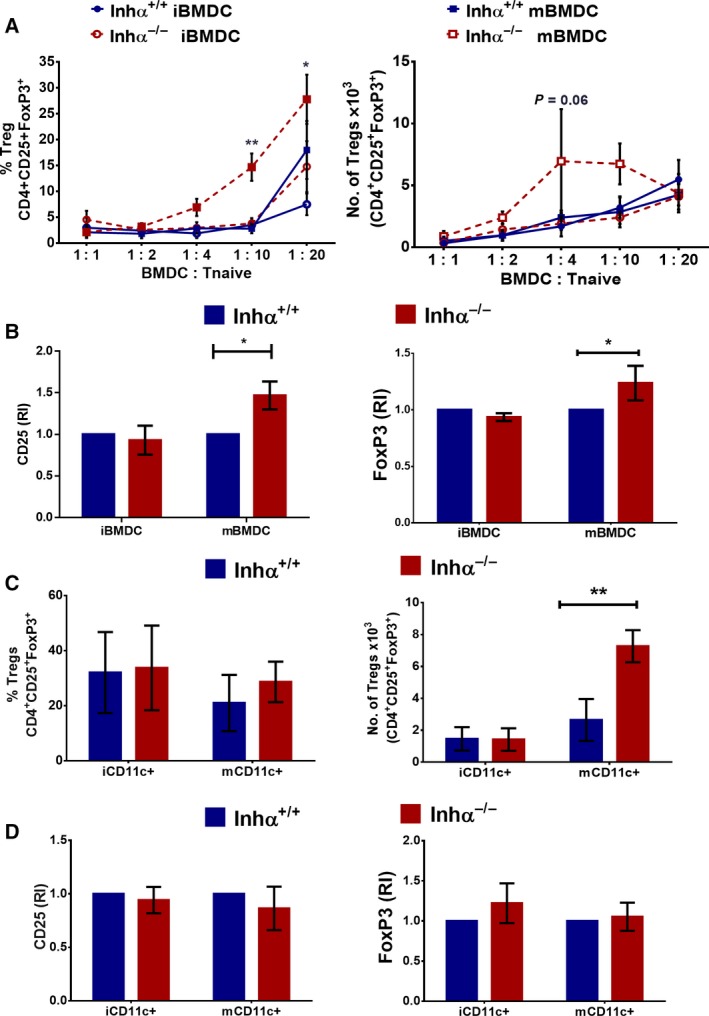
Inhibin controls DC‐dependent Treg cell induction *in vitro*. BMDC (A,B) or splenic and PLN CD11c^+^ DCs (C,D) were cocultivated with naïve T cells in presence of anti‐CD3 (0.1 μg·mL^−1^) and TGFβ (0.25 ng·mL^−1^). After 5 days, induction of Tre (CD4^+^CD25^+^FoxP3^+^) was evaluated. (A) Treg conversion from naïve T cells in the presence of wild‐type (WT) or Inhα^−/−^ iBMDCs or mBMDCs at different ratios. Graphs represent frequency (left) and total numbers (right) of Treg population. (B) CD25 (left) and FoxP3 (right) expression at induced Treg population are shown. (C) Treg conversion from naïve T cells in the presence of splenic and PLN iCD11c^+^ or mCD11c^+^ DCs, WT or Inhα^−/−^, at 1 : 5 ratio. Graphs represent frequencies (left) and total numbers (right) of Treg population. (D) CD25 (left) and FoxP3 (right) expression at induced Treg population as in (B). Bar graphs of CD25 and FoxP3 represent relative expression of MFI compared to Inhα^+/+^ mice. Mean ± SEM, *n* = 3. Statistical significance was determined by the two‐tailed paired Student's *t* test. *P ≤ 0.05, **P ≤ 0.01.

Naïve T cell differentiation towards an effector or regulatory phenotype requires several signals derived from the interaction between the T cell and the antigen presenting cell, including TCR–MHC, costimulation/coinhibition and cytokine mediated signals (reviewed in 21). Since MHC‐II, CD80 and PD‐L1 are altered in Inhα^−/−^ DCs, we next investigated whether inhibin expression by DCs could impact *in vitro* Treg conversion. In respect to this, we have previously reported that BMDCs express significant levels of inhibin A in response to LPS stimulation 33. As expected, Inhα^−/−^ did not produce detectable levels of inhibin A (Fig. S2). LPS‐stimulated Inhα^−/−^ BMDCs (mBMDCs) or non‐stimulated BMDCs (iBMDCs) were cocultured with naïve T cells in the presence of suboptimal concentrations of anti‐CD3 and TGFβ. We found that Inhα^−/−^ mBMDCs induced a higher percentage of CD25^+^FoxP3^+^ Tregs compared to Inhα^+/+^ mBMDCs (1 : 10 DC : T naïve ratio) (Fig. 3A). These differences may be in part explained by the upregulation of PD‐L1 and the ‘semi‐mature’ phenotype found in LPS‐stimulated Inhα^−/−^ BMDCs 33. The enhanced Treg conversion was accompanied by an increased CD25 and FoxP3 expression (Fig. 3B), suggesting that these induced Tregs might present an increased suppressive function 49. In this context, we have observed that total FoxP3^+^ Tregs purified from Inhα^−/−^ mice show increased suppressive activity towards polyclonally activated CD4^+^ T cells, in correlation with higher CD25 expression (data not shown). Moreover, LPS‐stimulated CD11c^+^ DCs (mCD11c^+^) from spleen and PLN of Inhα^−/−^ mice cocultured with naïve T cells, in the presence of suboptimal anti‐CD3 crosslinking and TGFβ, also induced a significantly higher generation of Tregs *in vitro* compared to their Inhα^+/+^ counterparts, indicating that Inhα^−/−^ DCs have an intrinsic enhanced capacity to promote peripheral T cell tolerance (Fig. 3C). No differences in the expression levels of CD25 or FoxP3 were observed between *in vitro* induced FoxP3^+^ Tregs in the presence of Inhα^−/−^ DCs compared to WT DCs (Fig. 3D).

### Inhα^−/−^ DCs enhance the induction of pTregs *in vivo*

To analyze the relevance of these findings *in vivo*, we used a strategy to directly deliver antigen to DCs, using anti‐DEC205‐OVA (α‐DEC‐OVA) DC targeting 23, 50 and evaluated the response of adoptively transferred OT‐II (OVA specific) TCR transgenic T cells. This system has been reported to generate either tolerogenic or immunogenic responses, depending on the adjuvant used during the α‐DEC205 targeting 23. Specifically, the use of CT as adjuvant induces effective Th1 and Th17 responses after intradermal immunization 23, while in the absence of adjuvant, α‐DEC205 antigen targeting promotes a tolerogenic response, by a mechanism that involves FoxP3^+^ Treg generation 51.

**Figure 4:**
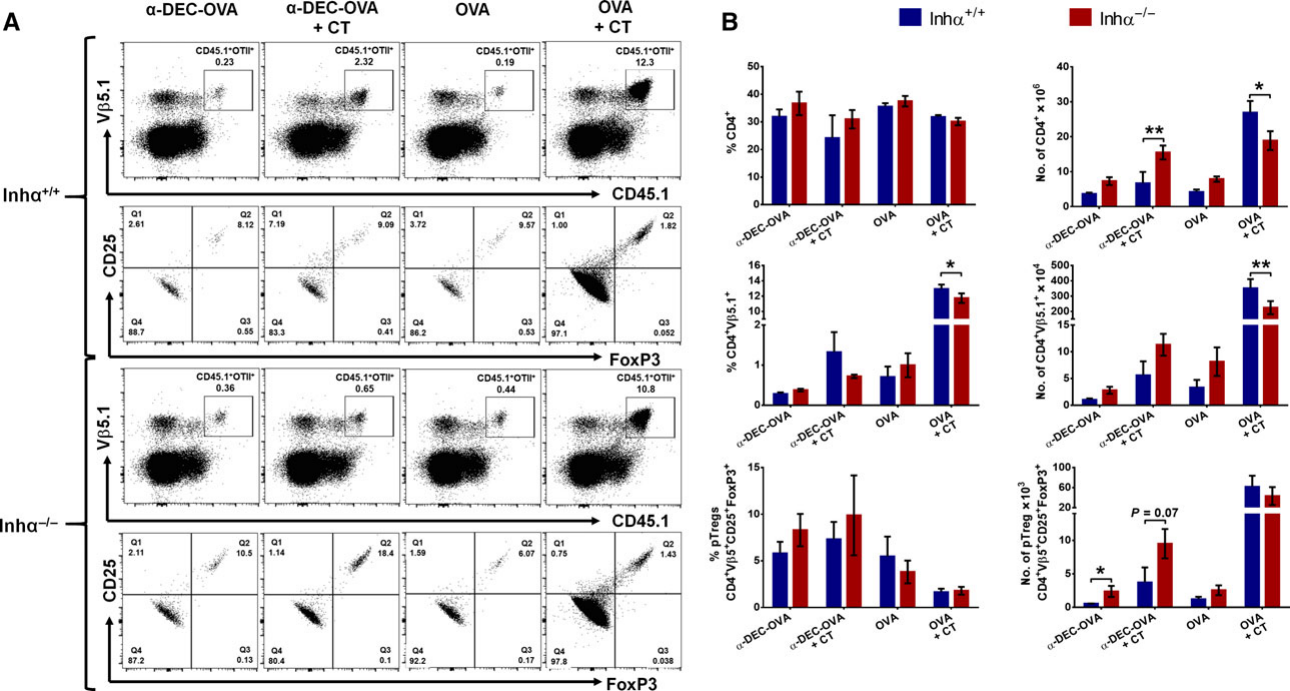
Antigen target of Inhα^−/−^ DCs through anti‐DEC205‐OVA induces an increased number of peripherally induced Tregs (pTregs) *in vivo*. OT‐II^+^CD45.1^+^ naïve T cells were transferred into Inhα^+/+^ or Inhα^−/−^ mice, and 24 h later mice were immunized intradermally in the ear, with anti‐DEC205‐OVA (α‐DEC‐OVA) or OVA, either with or without CT as adjuvant. Evaluation of pTregs was performed 7 days after immunization. (A) Representative dot plots of transferred cells (CD45.1^+^Vβ5^+^; top) and pTregs (CD25^+^FoxP3^+^; bottom) are shown for Inhα^+/+^ and Inhα^−/−^ receptor mice. (B) Percentage (left) and number (right) of CD4^+^ T cells (top), transferred cells (middle), and pTregs (bottom). Mean ± SEM, *n* = 3. Statistical significance was determined by two‐tailed unpaired Student's *t* test. *P ≤ 0.05, **P ≤ 0.01.

CD4^+^CD25^−^OT‐II^+^CD45.1^+^ naïve T cells were transferred intravenously to Inhα^−/−^ or Inhα^+/+^ CD45.2^+^ mice, and 24 h later they were immunized in the ear with soluble OVA or OVA‐targeted to DC through DEC205 (α‐DEC‐OVA), either with or without CT as adjuvant. Analysis of T cell responses in the dLN showed that immunization with OVA+CT resulted in a lower percentage and total numbers of transferred OVA‐specific (Vβ5^+^) CD4^+^ T cells in Inhα^−/−^ recipient mice compared to Inhα^+/+^ (Fig. 4A,B), suggesting that inhibins may regulate CD4^+^ T cell expansion, through the modulation of MHC‐II and costimulatory/coinhibitory molecules. Furthermore, we found a significant increase in the number of OT‐II^+^CD45.1^+^FoxP3^+^ pTregs in Inhα^−/−^ mice immunized with α‐DEC‐OVA compared to Inhα^+/+^, while Inhα^−/−^ mice immunized with α‐DEC‐OVA+CT showed a trend towards an increase in the number of pTregs compared to the Inhα^+/+^ counterparts, indicating that Inhα^−/−^ DCs are more prone to induce a tolerogenic response *in vivo* even in the presence of adjuvant.

The fact that PD‐L1 is upregulated in the absence of inhibins suggests that they could be a target to prevent tolerance induction in clinical protocols destined to boost the immune response, as PD‐1 blockage had been shown effective in anti‐tumor immunotherapy (reviewed in 52). In contrast, engagement of the PD‐L1/PD‐1 coinhibitory pathway is important for controlling several autoimmune diseases (reviewed in 53). Therefore, to understand how the expression of this coinhibitory molecule can be regulated is crucial for future clinical approaches.

In summary, our data demonstrate that inhibins regulate peripheral T cell tolerance by directly restraining pTreg generation *in vivo* through modulation of DC function. Our results are relevant for immunotherapy, identifying inhibins as new potential targets for immune intervention. By enhancing or blocking their effects, it would be possible promote immunogenic or tolerogenic responses in different pathological settings.

## Author contributions

GS conceived and designed the project. MF‐G, RO‐A and SO‐F performed the experiments. GS, LCB and MFG interpreted the data. GS and MFG wrote the paper. LB provided reagents. RO, SOF and LB critically reviewed the manuscript.

## Conflict of interest

The authors declare no conflict of interest.

## Supporting information


**Fig. S1.** *Ex vivo* analysis of DC subpopulations in MLN. Gating strategy to define DC subsets in MLN. Within the cells suspensions, CD19^−^CD3^−^TER119^−^NK1.1^−^ single live cells were selected for further analysis. The CD11c^hi^MHC‐II^Int^ population represents lymphoid rDCs and can be further divided into CD8α^+^ and CD8α^−^ DCs. CD11c^Int^MHC‐II^hi^ population represents mDCs, which can be further divided into CD103^+^CD11b^−^, CD103^+^CD11b^+^, CD11b^+^CD103^−^ and CD11b^−^CD103^−^.

**Fig. S2.** Inhibin A is produced by wild‐type DCs upon LPS stimulation but not by inhibin‐deficient (Inhα^−/−^) DCs. Time course of inhibin A from supernatants of wild‐type (Inhα^+/+^) or Inhα^−/−^ BMDC cultures were quantified by ELISA. Detection limit of the ELISA kit is represented by a blue line.
