# Supplementary figures and images for: Inhibins regulate peripheral regulatory T cell induction through modulation of dendritic cell function
Source: FEBS Open Bio. 2018 Dec 11;9(1):137–47. doi: 10.1002/2211-5463.12555 (PMC6325588; doi:10.1002/2211-5463.12555)

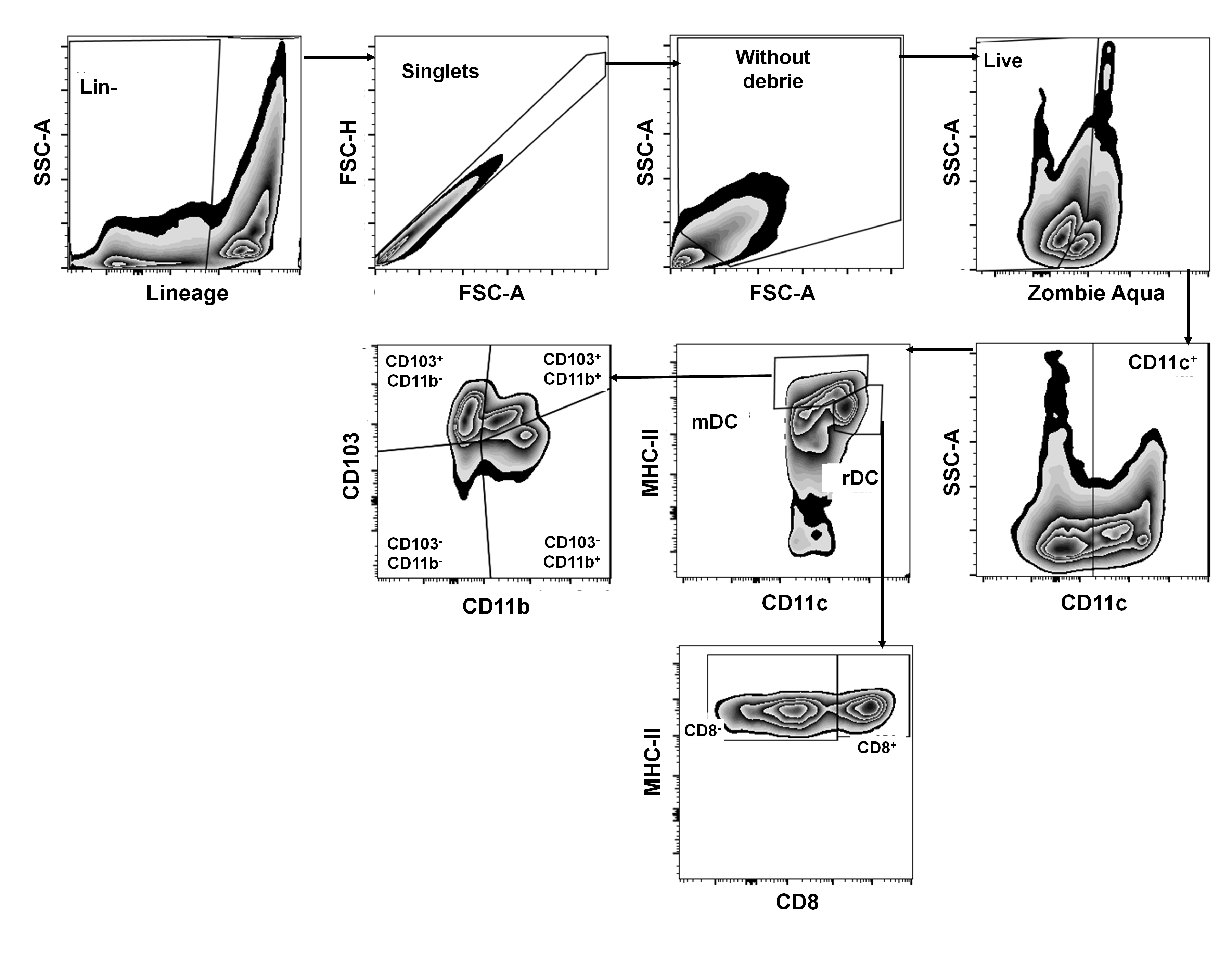

Supplement: Supplementary file 1 — Fig. S1. Ex vivo analysis of DC subpopulations in MLN. Gating strategy to define DC subsets in MLN. Within the cells suspensions, CD19−CD3−TER119−NK1.1− single live cells were selected for further analysis. The CD11chiMHC‐IIInt population represents lymphoid rDCs and can be further divided into CD8α+ and CD8α− DCs. CD11cIntMHC‐IIhi population represents mDCs, which can be further divided into CD103+CD11b−, CD103+CD11b+, CD11b+CD103− and CD11b−CD103−. [file FEB4-9-137-s001.tif]

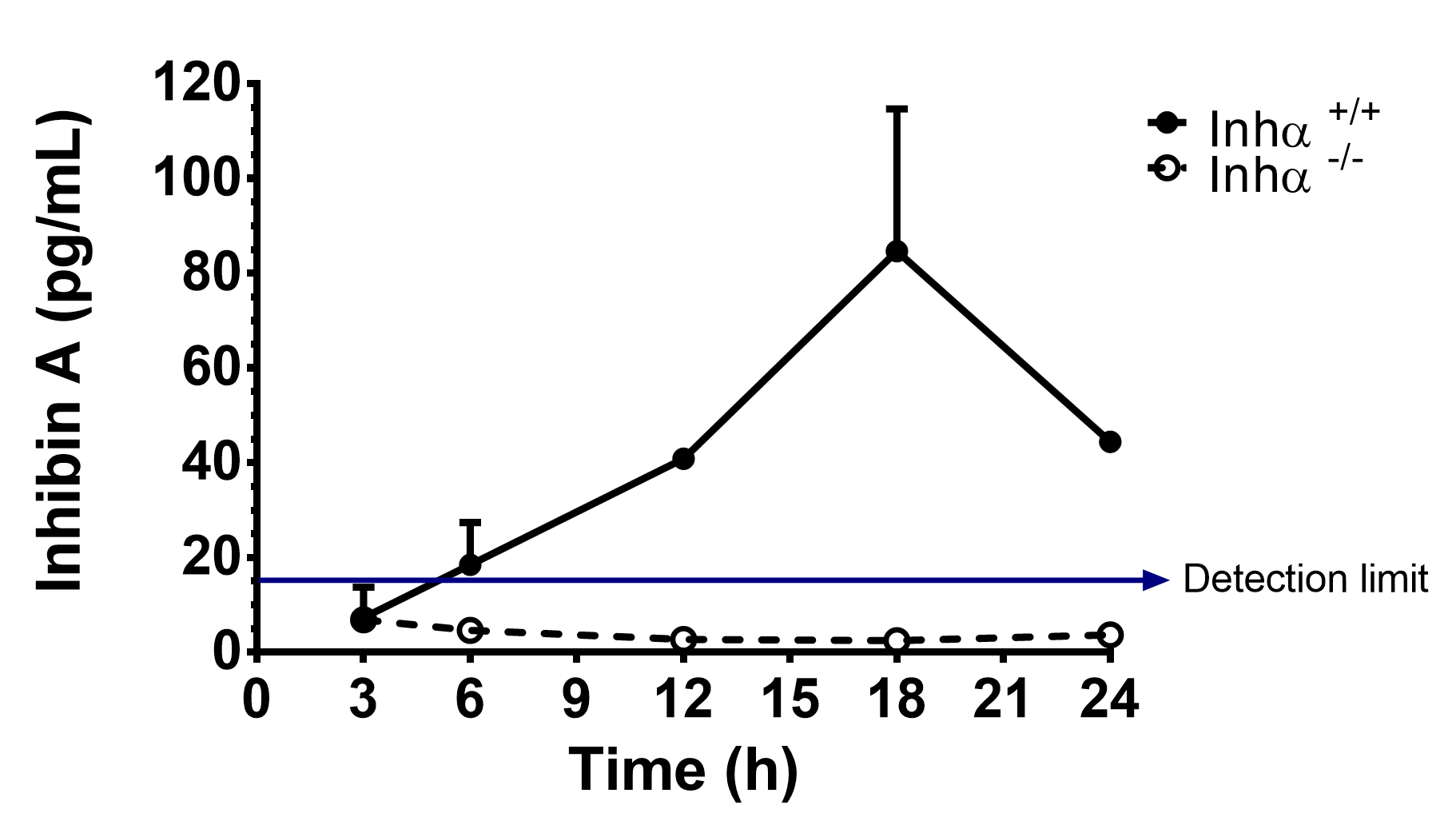

Supplement: Supplementary file 2 — Fig. S2. Inhibin A is produced by wild‐type DCs upon LPS stimulation but not by inhibin‐deficient (Inhα−/−) DCs. Time course of inhibin A from supernatants of wild‐type (Inhα+/+) or Inhα−/− BMDC cultures were quantified by ELISA. Detection limit of the ELISA kit is represented by a blue line. [file FEB4-9-137-s002.tif]
