# Supplementary material for: Inhibins regulate peripheral regulatory T cell induction through modulation of dendritic cell function
Source: FEBS Open Bio. 2018 Dec 11;9(1):137–47. doi: 10.1002/2211-5463.12555 (PMC6325588; doi:10.1002/2211-5463.12555)
Supplement: Supplementary file 3 [file FEB4-9-137-s003.docx]

**Supplementary Material**

**Supplementary Figure 1. *Ex vivo* analysis of DC subpopulations in MLN.** Gating strategy to define DC subsets in MLN. Within the cells suspensions, CD19^-^ CD3^-^ TER119^-^ NK1.1^-^ single live cells were selected for further analysis. The CD11c^hi^ MHC-II^Int^ population represents lymphoid resident DCs and can be further divided into CD8α^+^ and CD8α^-^ DC. CD11c^Int^ MHC-II^hi^ population represents migratory DCs, that can be further divided into CD103^+^CD11b^-^, CD103^+^CD11b^+^, CD11b^+^CD103^-^ and CD11b^-^CD103^-^.

**Supplementary Figure 2. Inhibin A is produced by wild type DCs upon LPS stimulation but not by Inhα**^-/-^ **deficient DCs**. Time course of Inhibin A from supernatants of wild type (Inhα^+/+^) or Inhibin deficient (Inhα^-/-^) BMDC cultures were quantified by ELISA. Detection limit of the ELISA kit is represented by a blue line.
